# Navigating the Anesthetic Challenges of Vertebral Defects, Anorectal Anomalies, Cardiac Anomalies, Tracheoesophageal Fistula (TEF)/Esophageal Atresia, Renal Anomalies, and Limb Abnormalities (VACTERL) Association: A Delicate Balancing Act

**DOI:** 10.7759/cureus.68797

**Published:** 2024-09-06

**Authors:** Fábio Costa, Maria Valentim, Carla Ferreira, Maria Santos

**Affiliations:** 1 Anesthesiology, Hospital de Braga, Braga, PRT

**Keywords:** anesthetic management, dopamine, imperforate anus, ketodex, newborn surgery, spontaneous ventilation, thoracoscopic, tracheoesophageal fistula, ultrasound-guided caudal block, vacterl association

## Abstract

Vertebral defects, anorectal anomalies, cardiac anomalies, tracheoesophageal fistula (TEF)/esophageal atresia, renal anomalies, and limb abnormalities (VACTERL) association is a rare congenital disorder presenting with a constellation of birth defects. The diagnosis is primarily clinical, and patients exhibit at least three of these anomalies. These patients' management involves a multidisciplinary approach tailored to the individual's condition. Anesthetic management is particularly challenging due to the diverse and complex anomalies. This article discusses the anesthetic management of a term newborn male (39 weeks, six days gestation, 3340 g) diagnosed with VACTERL association. The newborn was admitted to the neonatal intensive care unit (NICU) and scheduled for the surgical repair of TEF and derivative colostomy on the second day of life. To mitigate the risk of air leak and abdominal distension from positive pressure ventilation, a derivative colostomy was performed first under regional anesthesia preserving spontaneous ventilation. To achieve that, the patient was sedated with ketamine and dexmedetomidine, and an ultrasound-guided single-shot caudal block with ropivacaine was performed. Post-abdominal decompression, general anesthesia was induced, and intubation was managed via videolaryngoscopy. Thoracoscopic TEF repair required several pauses for ventilation and hemodynamic optimization. Dopamine was administered intraoperatively for blood pressure support. The newborn was extubated and started on enteral feeding by the seventh postoperative day, progressing well by the time of discharge. In this case, a derivative colostomy before TEF repair avoided positive pressure ventilation complications. Ultrasound-guided caudal block provided effective regional anesthesia with high success rates. Ketamine and dexmedetomidine offered balanced sedation with minimal respiratory compromise. Dopamine was used effectively to maintain adequate perfusion, monitored with invasive blood pressure and cerebral oximetry. Anesthetic management of newborns with VACTERL association undergoing simultaneous repair of TEF and anal atresia demands meticulous and tailored planning to address the specific needs and minimize associated risks. This case highlights the importance of comprehensive anesthetic management and its impact on the patient's outcome.

## Introduction

Vertebral defects, anorectal anomalies, cardiac anomalies, tracheoesophageal fistula (TEF)/esophageal atresia, renal anomalies, and limb abnormalities (VACTERL) association is a rare congenital disorder characterized by a specific set of birth defects that occur commonly together without an established cause. Though there are several syndromes with overlapping features, its occurrence is largely sporadic. There is no identified common genetic defect, and its cause is thought to be multifactorial, with genetic and environmental contributing factors. Due to the number and multisystem organs affected, it is thought that a "developmental field defect" occurs during blastogenesis (2-4 weeks of gestation), where abnormal structures are derived from the embryonic mesoderm. There are no validated diagnostic criteria published. VACTERL association is a diagnosis of exclusion, and it is based on the presence of three or more congenital defects and the absence of clinical or laboratory evidence for similar overlapping conditions [1]. 

The prevalence of the VACTERL association is estimated to be between one in 10,000-40,000 live births, with a slight male predominance [1]. TEF is observed in approximately 50-80% of affected individuals. After birth, these newborns are noted to have excessive salivation, requiring repeated suctioning or choking on the first feeding attempts. A definite diagnosis of TEF will be established when the gastric tube does not progress to the stomach and the chest/abdomen X-ray reveals a coiled tube in the esophageal position, along with a gastric air bubble [2]. Surgical intervention is typically required within the first few days of life and consists of disconnection of the TEF, closure of the tracheal defect, and primary anastomosis of the esophagus. In situations where there is a long gap between the esophagus ends, the anastomosis should be delayed. Postoperative complications can arise, including fistula recurrence, reactive airway disease, and gastroesophageal reflux [3].

Imperforate anus, or anal atresia, is a component of anorectal malformations (ARM) that occurs in approximately 55-90% of cases [4]. This condition is frequently identified shortly after birth, either through standard newborn examinations or due to difficulties encountered when attempting to take the infant's rectal temperature [5]. 

Diagnosis of VACTERL association is primarily clinical, based on the presence of the hallmark anomalies. Its management involves a multidisciplinary approach tailored to the specific anomalies present in each individual, with the aim of addressing both immediate- and long-term health challenges [6].

Anesthetic management of patients with VACTERL association presents a significant challenge due to the diverse and complex anomalies associated with the condition. Each component of the VACTERL acronym can contribute to unique difficulties during anesthesia, necessitating a highly individualized and cautious approach. Patients with VACTERL association often have TEF and esophageal atresia, which complicates airway management. These anomalies are associated with preoperative aspiration episodes which aggravate respiratory conditions. Intubation and ventilation often require the use of advanced airway techniques and equipment [7]. Cardiac defects, which are common in VACTERL association, further complicate anesthetic management. These defects can range from ventricular septal defects to more complex congenital heart diseases, affecting hemodynamic stability [8]. Renal anomalies, including agenesis and dysplasia, pose additional risks during anesthesia. Impaired renal function can affect the pharmacokinetics and pharmacodynamics of anesthetic agents, needing careful selection and dosing of drugs [9]. Vertebral defects and limb abnormalities can influence positioning and access during surgery.

Mortality data related to the VACTERL association can be challenging to quantify due to the variability in the severity and combination of congenital anomalies present in affected individuals. Mortality rates are influenced by several factors, including the presence of severe cardiac, renal, or respiratory defects, which can significantly impact survival, especially in the neonatal period. Studies suggest that infants with multiple or severe anomalies, particularly those requiring complex surgical interventions, face higher mortality risks. However, advancements in prenatal diagnosis, neonatal care, and surgical techniques have improved survival rates over time. Despite these improvements, the mortality rate remains significant for severe cases, underscoring the importance of early detection and comprehensive medical care. Accurate and detailed data collection is crucial for understanding the full impact of the VACTERL association on mortality and for developing strategies to improve patient outcomes [10].

In this article, we describe the anesthetic management of a newborn diagnosed with VACTERL association who was scheduled for correction of TEF/esophageal atresia and anal imperforation on the second day of life.

## Case presentation

**Figure 1:**
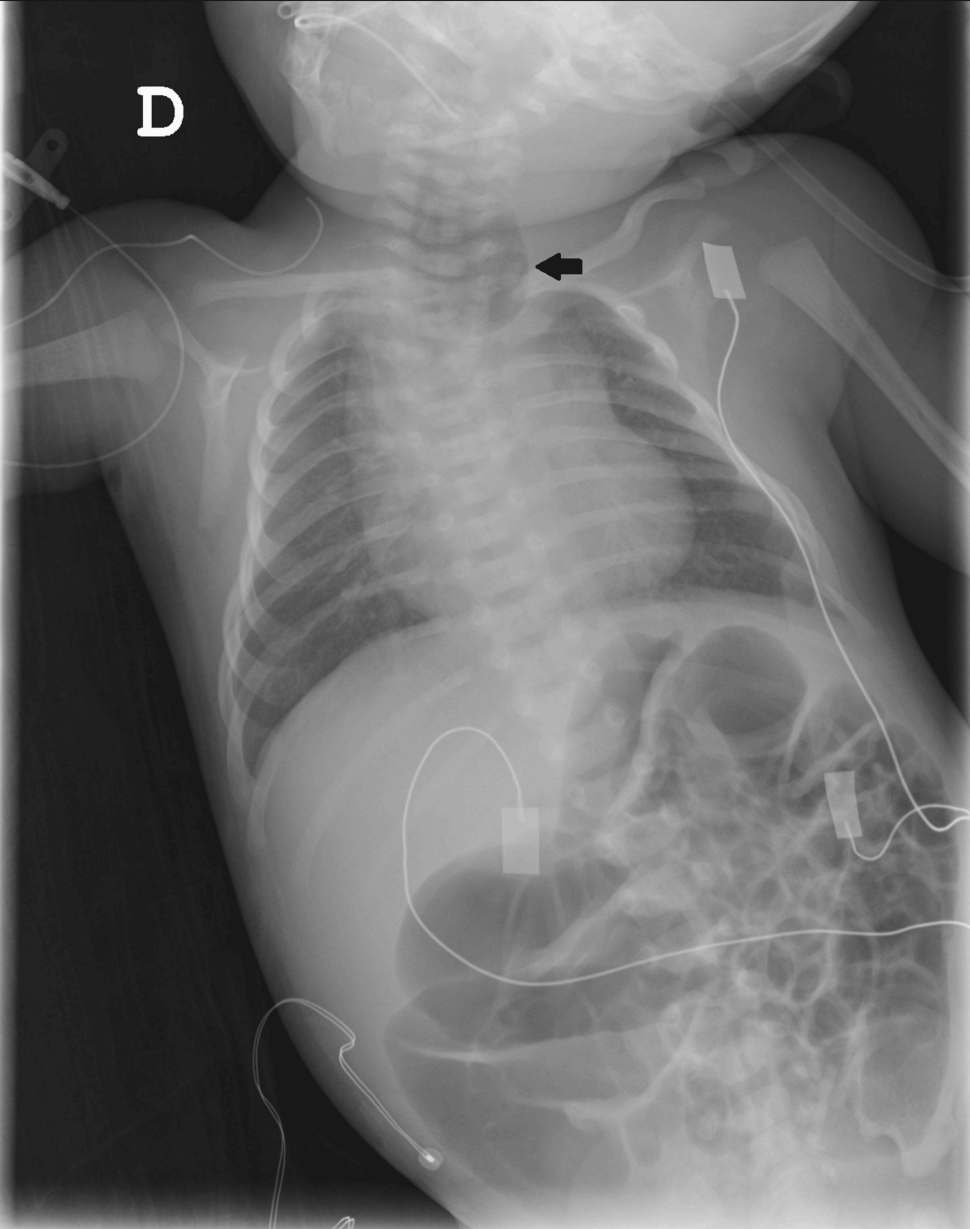
Thoracic radiograph Frontal chest radiograph demonstrating an esophageal pouch (black arrow) and air in the stomach and intestine, compatible with esophageal atresia with distal fistula.

We describe the anesthetic management of a neonate (39 weeks and six days) male, ASA III, weighing 3340 g diagnosed with VACTERL association (encompassing anal atresia, TEF, renal abnormalities, and polydactyly). Renal agenesia was diagnosed during prenatal ultrasound, and TEF and anal atresia were diagnosed after birth as the gastric tube could not be passed into the stomach. A thoracic X-ray (Figure 1) unveiling the coiled gastric tube in the esophagus and a gastric air bubble confirmed the diagnosis.

After birth, the newborn was optimized in the neonatal intensive care unit (NICU) and was clinically and hemodynamically stable, comfortable, and in spontaneous ventilation in room air. The patient was proposed for the surgical repair of TEF (type C) and imperforate anus on the second day of life. The case was discussed with the surgical team, and the concern of air leak and gastric and bowel distension was put into account if positive pressure ventilation was applied. Thus, it was agreed to perform the derivative colostomy with spontaneous ventilation under regional anesthesia first and then proceed to thoracoscopic TEF correction under mechanical ventilation. 

**Figure 2:**
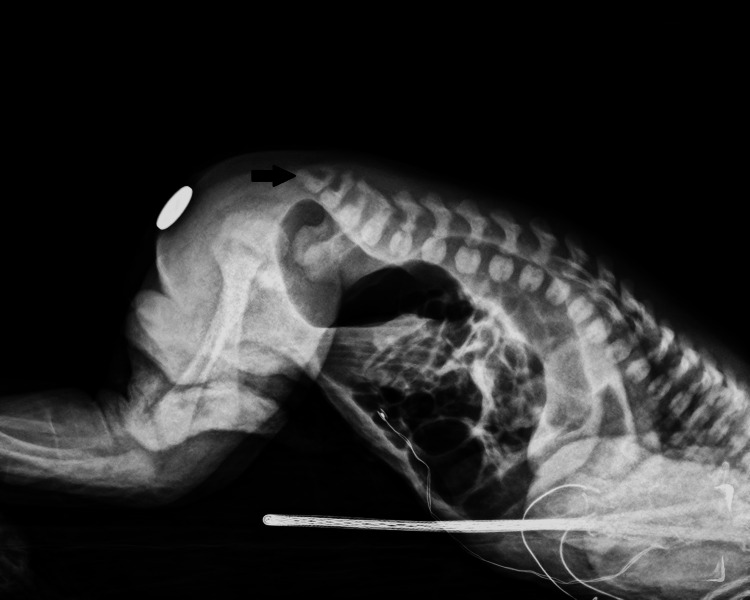
Spine radiograph Spine X-ray showing an apparently well-formed sacrum (black arrow), without pre-sacred masses visualized.

A comprehensive preoperative evaluation was conducted to assess the patient's readiness for the planned surgical procedure. The preoperative echocardiogram revealed the presence of mild tricuspid insufficiency and mild pulmonary insufficiency. Additionally, an interatrial communication was detected. The lung ultrasound examination and spine X-ray (Figure 2) showed no abnormalities. The transfontanellar ultrasound also revealed no abnormalities. The blood gas analysis preoperatively indicated a mild metabolic acidosis. Despite this finding, the patient did not present with severe acid-base disturbances that would contraindicate proceeding with surgery. Routine blood analysis (Table 1) did not show any significant abnormalities. All measured electrolytes were within normal ranges, indicating stable metabolic and renal function.

**Table 1:** Laboratory test results IPF: immature platelet fraction; MCH: mean corpuscular hemoglobin; MCHC: mean corpuscular hemoglobin concentration; MCV: mean corpuscular volume; RDW: red blood cell distribution width

Test name	Result	Normal range	Unit
Hematocrit	50.5	48-69	%
Hemoglobin	17.4	15.0-24.0	g/dL
Erythrocytes	4.6	4.0-6.6	10^6^/uL
MCV	109.8	84-95	fL
MCH	37.8	28-34	pg
MCHC	34.5	29-36	g/dL
RDW	16.0	15.5-20	%
Erythroblasts	3.2	0-10	/100WBC
White blood cells	25.0	9.4-34.0	10^3^/uL
Neutrophils	14.9	7.0-14.5	10^3^/uL
Eosinophils	0.5	0.2-1.9	10^3^/uL
Basophils	0.2	0.0-0.1	10^3^/uL
Lymphocytes	6.7	2.0-7.3	10^3^/uL
Monocytes	2.3	0.2-1.8	10^3^/uL
Immature granulocytes	0.6		10^3^/uL
Platelets	234	150-350	10^3^/uL
IPF	2.0	1.1-7.7	%
Calcium	10.8	8.3-10.6	mg/dL
C-reactive protein	<0.5	<3.0	mg/L

The patient was monitored according to ASA standards (oximetry, capnography, electrocardiogram, invasive blood pressure, and core temperature) as well as with cerebral oximetry (INVOS™). The patient was sedated with an initial bolus of ketamine (2 mg) and dexmedetomidine (2 mcg) maintaining spontaneous ventilation with a nasal cannula at 1 liter per minute. Top-up boluses were administered to a total of 5 mg of ketamine and 5 mcg of dexmedetomidine. An ultrasound-guided single-shot caudal block was performed, and 7 mg ropivacaine 0.2% was administered through a 25G Epican® needle. After derivative colostomy and abdominal decompression, general anesthesia was induced, and the patient was intubated in spontaneous ventilation with a 2.5 mm internal diameter (ID) cuffed endotracheal tube by videolaryngoscopy. During the thoracoscopic TEF repair, anesthesia was maintained with fentanyl (bolus of 3 mcg), ketamine and dexmedetomidine (perfusion of dexmedetomidine 10 mcg/mL+ketamine 1 mg/mL at a flow rate of 3.5 mL/h), and rocuronium (bolus of 1.5 mg). The intraoperative period elapsed with the need to pause surgery several times, to optimize ventilation and hemodynamics. It was necessary to change the endotracheal tube for one cuffed with 4.0 mm ID to allow the optimization of ventilation. Dopamine was initiated during surgery at 5 mcg/kg/min to a maximum of 10 mcg/kg/min. Fluid management was based on a balanced solution with 1% dextrose and 10 mL/kg bolus as needed. 

The neonate was transferred to the NICU immediately after surgery and was kept sedated under mechanical ventilation. The patient was electively extubated on the eighth day after surgery. The newborn presented a dehiscence of the thoracic wound and remained in the NICU for 28 days. After this period, the patient was discharged home.

## Discussion

VACTERL association

VACTERL association is a non-random combination of birth defects that affects multiple anatomical structures. Its diagnosis is most commonly established after birth, but prenatal identification of anomaly patterns is possible. In our case, renal agenesia was diagnosed during the prenatal period, and the anal atresia, TEF, and polydactyly were diagnosed on the first day of life. This patient was promptly referred for surgical management following diagnosis at birth, highlighting the importance of timely intervention to improve outcomes. 

Airway and ventilation management

One of the main concerns in approaching this case was the ventilation management during surgical correction. Airway and ventilation management in TEF repair is challenging. A thoughtful approach with alternative options readily available, as well as an understanding of the merits and pitfalls of each strategy, is important. High-level evidence on how best to manage the airway and ventilation in TEF is scarce or non-existent. Several factors must be considered in the airway and ventilation management plan: TEF size and location, the clinical respiratory status of the neonate, the planned surgical approach, and the necessary patient positioning. The classical technique is to pass the endotracheal tube tip beyond the fistula (but proximal to the carina) to prevent insufflation of the stomach. If this is the option chosen, it is important to be aware of the potential movements of the endotracheal tube during positioning and surgical manipulation in a very small trachea [11]. 

In this case, the concomitant existence of an imperforate anus means that there was a dead-end gastrointestinal system. The entry of air into the gastrointestinal system would increase intra-abdominal pressure and, concomitantly, intrathoracic pressure, which would make ventilation difficult or impossible.

For the success of ventilatory management, dialogue between the anesthesia and pediatric surgery teams was essential. The decision to perform a derivative colostomy first under spontaneous ventilation allowed the avoidance of positive pressure ventilation in a patient with TEF and imperforate anus. 

This case stands out because the colostomy was performed with spontaneous ventilation, thus avoiding mechanical ventilation of a patient with TEF and imperforate anus, which would cause air to enter a dead-end gastrointestinal system, making ventilation difficult or impossible and increasing the risk of aspiration of gastric content through the fistula.

Anesthesia technique

Derivative colostomy was performed with the use of ultrasound-guided caudal block and sedation with ketamine and dexmedetomidine.

Ultrasound-guided caudal block has several advantages over landmark-based methods. Although the landmark approach has a good success rate (above 96%) [12], the use of ultrasound appears to increase the success rate of a first puncture compared to the traditional approach [13]. Real-time ultrasound monitoring of local anesthetic spread also allows for the visual confirmation of correct placement. Abnormalities of sacral formation such as sacral dysgenesis can also be elucidated using ultrasound scanning prior to block administration as not all infants with sacral abnormalities have associated cutaneous stigmata. Despite these notable advantages, so far there has not been any decrease in morbidity and mortality found when using an ultrasound technique [14].

The use of ketamine and dexmedetomidine for sedation and analgesia proved to be beneficial, as it allowed for a balanced and titratable sedation level while maintaining spontaneous ventilation. The ketamine and dexmedetomidine combination also provided effective sedation with hemodynamic stability. Ketamine is an N-methyl-D-aspartate (NMDA) receptor antagonist and provides effective analgesia and sedation while preserving spontaneous ventilation and airway reflexes. It is known for its sympathomimetic effects and can help maintain hemodynamic stability, which is particularly important in high-risk pediatric patients. Dexmedetomidine, an alpha-2 adrenergic agonist, produces sedation, anxiolysis, and analgesia. It has a unique profile with minimal respiratory depression, making it an attractive choice in cases where maintaining spontaneous ventilation is preferred. The combination of ketamine and dexmedetomidine allows for a balanced anesthetic effect, providing adequate sedation and analgesia without causing significant respiratory compromise. By avoiding the use of volatile agents and opioids, the risks of respiratory depression and airway complications were minimized, which was crucial in this case [15].

Cardiovascular considerations

Cardiac anomalies are common in VACTERL association and can range from minor defects to complex congenital heart diseases [7], significantly impacting anesthetic management. The preoperative echocardiogram of this patient revealed the presence of mild tricuspid and pulmonary insufficiency and atrial septal defect. It was necessary to use vasopressors in the intraoperative period to maintain blood pressure targets sufficient to maintain organ perfusion. Organ perfusion was monitored through an electrocardiogram with ST-T analysis, cerebral oximetry, and urinary output. Dopamine was used as it was the vasopressor which the anesthetic team was most comfortable with. Despite this, the use of norepinephrine in comparison with dopamine in neonatal patients has been investigated, and it seems to have comparable efficacy when used for the treatment of neonatal and pediatric septic shock [16]. 

## Conclusions

The anesthetic management of a newborn with VACTERL association undergoing simultaneous repair of TEF and imperforate anus requires careful planning, a multidisciplinary approach, and the use of balanced anesthetic techniques to optimize outcomes. This case exemplifies the importance of tailoring anesthetic strategies to the specific needs and risks associated with congenital anomalies. This case stands out because the colostomy was performed with spontaneous ventilation, thus avoiding mechanical ventilation of a patient with TEF and imperforate anus, which would cause air to enter a dead-end gastrointestinal system, making ventilation difficult or impossible and increasing the risk of aspiration of gastric content through the fistula.

## References

[1] Solomon BD (2011). VACTERL/VATER association. Orphanet J Rare Dis.

[2] Spitz L (2007). Oesophageal atresia. Orphanet J Rare Dis.

[3] Raam MS, Pineda-Alvarez DE, Hadley DW, Solomon BD (2011). Long-term outcomes of adults with features of VACTERL association. Eur J Med Genet.

[4] Källén K, Mastroiacovo P, Castilla EE, Robert E, Källén B (2001). VATER non-random association of congenital malformations: study based on data from four malformation registers. Am J Med Genet.

[5] Rittler M, Paz JE, Castilla EE (1996). VACTERL association, epidemiologic definition and delineation. Am J Med Genet.

[6] Solomon BD, Raam MS, Pineda-Alvarez DE (2011). Analysis of genitourinary anomalies in patients with VACTERL (vertebral anomalies, anal atresia, cardiac malformations, tracheo-esophageal fistula, renal anomalies, limb abnormalities) association. Congenit Anom (Kyoto).

[7] (2014). Anaesthesia recommendations for patients suffering from VACTERL association. https://www.orphananesthesia.eu/en/rare-diseases/published-guidelines/vacterl-association/174-vacterl-association/file.html.

[8] Cunningham BK, Hadley DW, Hannoush H (2013). Analysis of cardiac anomalies in VACTERL association. Birth Defects Res A Clin Mol Teratol.

[9] Cunningham BK, Khromykh A, Martinez AF, Carney T, Hadley DW, Solomon BD (2014). Analysis of renal anomalies in VACTERL association. Birth Defects Res A Clin Mol Teratol.

[10] Stein RA (2007). Smith's recognizable patterns of human malformation, 6th edition. Arch Dis Child.

[11] Ho AM, Dion JM, Wong JC (2016). Airway and ventilatory management options in congenital tracheoesophageal fistula repair. J Cardiothorac Vasc Anesth.

[12] Kao SC, Lin CS (2017). Caudal epidural block: an updated review of anatomy and techniques. Biomed Res Int.

[13] Ahiskalioglu A, Yayik AM, Ahiskalioglu EO (2018). Ultrasound-guided versus conventional injection for caudal block in children: a prospective randomized clinical study. J Clin Anesth.

[14] Suresh S, Long J, Birmingham PK, De Oliveira GS Jr (2015). Are caudal blocks for pain control safe in children? An analysis of 18,650 caudal blocks from the Pediatric Regional Anesthesia Network (PRAN) database. Anesth Analg.

[15] Vila Moutinho Tavares S, Tavares JC, Borges Marques J, Teixeira de Figueiredo J, Passos de Souza RL (2023). Ketamine-dexmedetomidine combination for sedation in pediatric major surgery in a low-income country. Paediatr Anaesth.

[16] Wen L, Xu L (2020). The efficacy of dopamine versus epinephrine for pediatric or neonatal septic shock: a meta-analysis of randomized controlled studies. Ital J Pediatr.

